# Development of a HPLC fluorometric method for the quantification of enfuvirtide following in vitro releasing studies on thermosensitive in situ forming gel

**DOI:** 10.1007/s13346-023-01344-5

**Published:** 2023-04-29

**Authors:** Huanhuan Li, Qonita Kurnia Anjani, Mary B. McGuckin, Achmad Himawan, Mingshan Li, Ryan F. Donnelly

**Affiliations:** 1 School of Pharmacy, Queen’s University Belfast, 97 Lisburn Road, Belfast, BT9 7BL UK; 2 Department of Pharmaceutical Science and Technology, Faculty of Pharmacy, Universitas Hasanuddin, Makassar, 90245 Indonesia; 3 Fakultas Farmasi, Universitas Megarezky, Jl. Antang Raya No. 43, Makassar, 90234 Indonesia

**Keywords:** Enfuvirtide, Peptide, HPLC, Fluorescence detector, Intradermal delivery

## Abstract

Due to the presence of peptidase and protease in the gastrointestinal tract, peptides are subjected to digestion and inactivation when administrated orally. To avoid degradation and maintain the desired efficacy of peptide drugs, there is a demand to develop transdermal and intradermal delivery systems. This requires efficient and specific analytical methods to separate and quantify the peptide drugs from the formulation and the skin matrix in the early stages of pharmaceutical development. A high-performance liquid chromatography (HPLC) system equipped with a fluorometric detector was used to quantify enfuvirtide, which is the first fusion inhibitor for HIV treatment. The HPLC method was developed and validated according to the ICH Q2(R1) guidelines. The viability of the method was demonstrated during in vitro studies, where samples were analysed following intradermal administration of a thermosensitive in situ forming gel. Compared with previously reported methods, this assay proved efficient, sensitive and accurate, with a detection limit of 0.74 μg/mL and a run time of 9 min, mitigating the use of any internal standards and detergents. The addition of an organic solvent to the samples successfully solved the problem of low recovery caused by the adsorption of the drug to the plastic consumables in the sample treatment process. The amount of enfuvirtide releasing from the in situ gel through skin after 7 hours was 16.25 ± 7.08 μg, which was significantly lower than the reconstituted FUZEON^®^ itself (26.68 ± 10.45 μg), showing a longer release profile. The results may be beneficial as a constructive input for future enfuvirtide quantification within a preclinical setting through in vitro release studies across the skin.

**Graphical Abstract:**

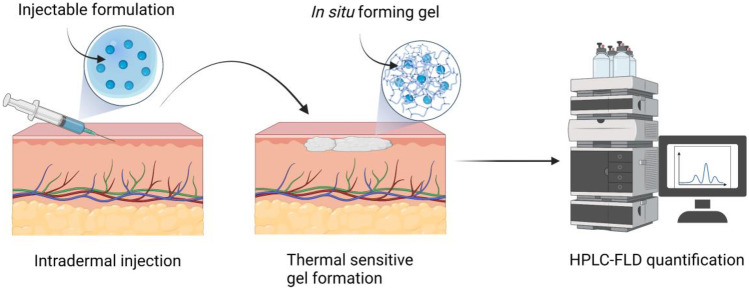

## Introduction

Compared with proteins, peptides provide similar therapeutic benefits, but with improved tissue penetration, reduced immunogenicity and lower drug production costs [1–5]. However, their low oral bioavailability, poor plasma stability and short circulation have presented challenges for their delivery [6]. In order to protect peptides against proteolytic degradation from oral administration, transdermal drug delivery (TDD) has been considered a prospective administration route for peptide drugs [7–9]. Due to their physicochemical properties such as molecular weight greater than 500 Da and varied lipid-water partition coefficient, no peptide product applied topically is available on the market yet. The main routes for peptides to be delivered are subcutaneous and intramuscular injection. Additionally, peptides are characterised with short half-lives; thus, frequent injections are necessary in clinical use, inducing undesirable injection site reactions (ISRs) on patients [1]. Therefore, intradermal platforms for prolonged release of peptide drugs are imperative. Apart from strategies such as implants and crosslinked gels, thermosensitive in situ forming gels can be employed to achieve a stable plasma concentration within the therapeutic window. Formulated with biocompatible excipients, the peptide drug is incorporated into an injectable biomaterial and undergoes in situ self-gelation after being injected into the skin. Accordingly, simple and sensitive quantification methods for release studies on in situ gels containing peptides should also be developed [6, 10–12].

**Fig. 1:**
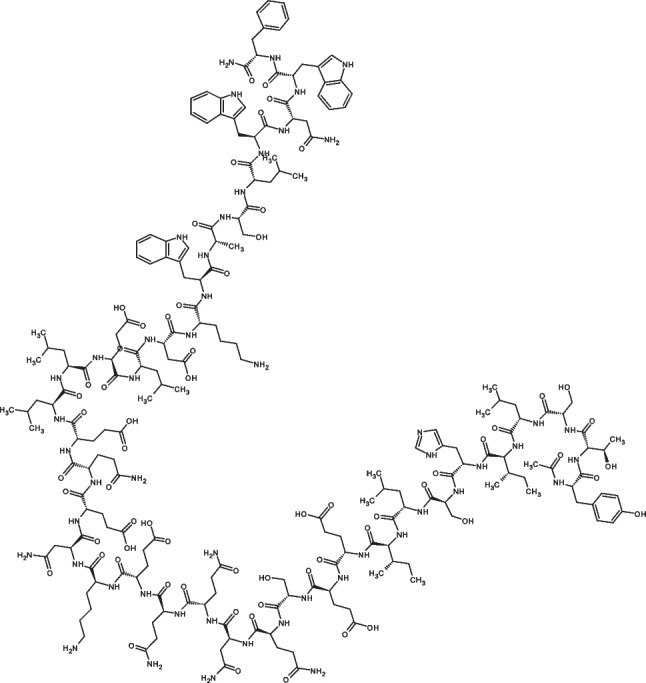
Chemical structure of enfuvirtide [14]

As a HIV-1 fusion inhibitor, enfuvirtide (FUZEON^®^) is a synthetic 36-amino acid peptide (shown in Fig. 1). Whilst demonstrating remarkable antiviral activity compared with an optimised background (OB) antiretroviral regimen alone, the application of enfuvirtide was largely confined by its side effects. Currently, enfuvirtide can only be given via subcutaneous injection [13–16]. Due to the rapid metabolism by peptidase, twice daily administration is required with a dosage of 90 mg per injection, which considerably increases the ISRs in patients over a long period of treatment [17]. To improve compliance, less-painful and long-acting transdermal delivery methods have been investigated to deliver enfuvirtide, such as Biojector^®^, ultrasound transducer patch and polymer-lipid hybrid nanoparticles [18–22].

Chromatographic methods have previously been established for the determination of enfuvirtide in plasma using mass spectrometry (MS) and fluorescence detectors (FLD), where costly internal standards and complex sample preparations were required [23–26]. As a result of the electrostatic interactions between amino acids and the silanol groups on glass surfaces, and nonpolar amino acid interactions with the hydrophobic surface of poly (propylene) (PP) containers, adsorption of the peptide to the container remains a challenge in chromatography methods [27, 28]. Moreover, it has been reported by D’Avolio et al. [23] and Lawless et al. [29] that specific centrifuge tubes made of poly(tetrafluoroethylene) (PTFE) were imperative during processing procedures of enfuvirtide. The addition of displacement agents, such as structural analogues or protein-rich solutions that compete with the analyte for surface binding sites, has been advised as a solution for adhesion, which, to some extent, increases analysis complexity [6].

Herein, a simple, accurate and sensitive enfuvirtide HPLC fluorometric quantitative method was developed and validated according to ICH guidelines Q2(R1). The chromatographic conditions in this method were capable of separating the peptide from the skin matrix specifically. The method was then successfully employed to quantify enfuvirtide in the release studies of the injectable in situ forming gel. This work may be beneficial for researchers who work with peptides and intend to deliver the peptides intradermally.

## Materials and methods

### Chemicals and reagents

Enfuvirtide (Fuzeon^®^) was purchased from Roche Co. Ltd. (Basel, Switzerland). Poloxamer 188 (Kolliphor^®^ P 188), poloxamer 407 (Kolliphor^®^ P 407), acetonitrile (ACN, HPLC-grade) and phosphate buffered saline (PBS) tablets were obtained from Sigma-Aldrich (Dorset, UK). Neonatal porcine skin was excised from piglets and stored at −20 °C before use. HPLC-grade water was purified by the Elga Option Purelab water purification system (Elga LabWater, High Wycombe, UK).

### Chromatographic conditions

The analysis was performed on an Agilent 1260 Infinity II LC system (Agilent Technologies UK Ltd., Stockport, UK) equipped with 1260 Quat Pump G7111B, a G7129A Vialsampler and a G7121A fluorometric detector (excitation wavelength 280 nm, emission wavelength 350 nm). The fluorescence detector gain was set at 10 with an attenuation setting of 100. The ODS-3 analytical column (250 mm × 4.6 mm internal diameter, 5 μm packing; Inertsil^™^, GL Sciences, JP; InertClone^™^, Phenomenex, Torrance, USA) protected by a precolumn (SecurityGuard^™^ Guard Cartridge Kit, Phenomenex, Torrance, USA), was used to separate and quantify enfuvirtide at 25 °C. The mobile phase was composed of solvent A (deionised water with 0.1% v/v phosphoric acid, pH 2.5) and solvent B (ACN). A gradient with a flow rate of 1 mL/min was applied in the following way: 35–45% B (0–3 min), 45–75% B (3–5.5 min), 75% B (5.5–7 min), 75–35% B (7–9 min), as shown in Table 1. The injection volume was 30 µL, followed by six needle washes.

**Table 1:** Chromatographic condition (gradient)

**Time (min)**	**% Solvent A**	**% Solvent B**	**Flow (mL/min)**
0.0	65.0	35.0	1.0
3.5	55.0	45.0	1.0
5.5	75.0	75.0	1.0
7.0	35.0	35.0	1.0

### Stock, calibration standard and quality control solution preparation

A 1-mg/mL standard stock solution of enfuvirtide was prepared by dissolving 10 mg enfuvirtide powder in PBS (pH 7.4, 10 mM) in a 10-mL volumetric flask and diluting appropriately. For the preparation of the calibration curve, mobile phase (water:acetonitrile = 50:50, v/v) was used as the diluent and the standard solution (STD) was prepared by serial gradient dilution of the stock solution to obtain the following concentrations: 25, 12.5, 6.25, 3.12, 1.56, 0.78, 0.39 and 0.19 µg/mL. Quality control (QC) samples, low QC (1.56 µg/mL), mid QC (6.25 µg/mL) and high QC (25 µg/mL), were prepared with the same procedure as the STD.

### Sample preparation

**Fig. 2:**
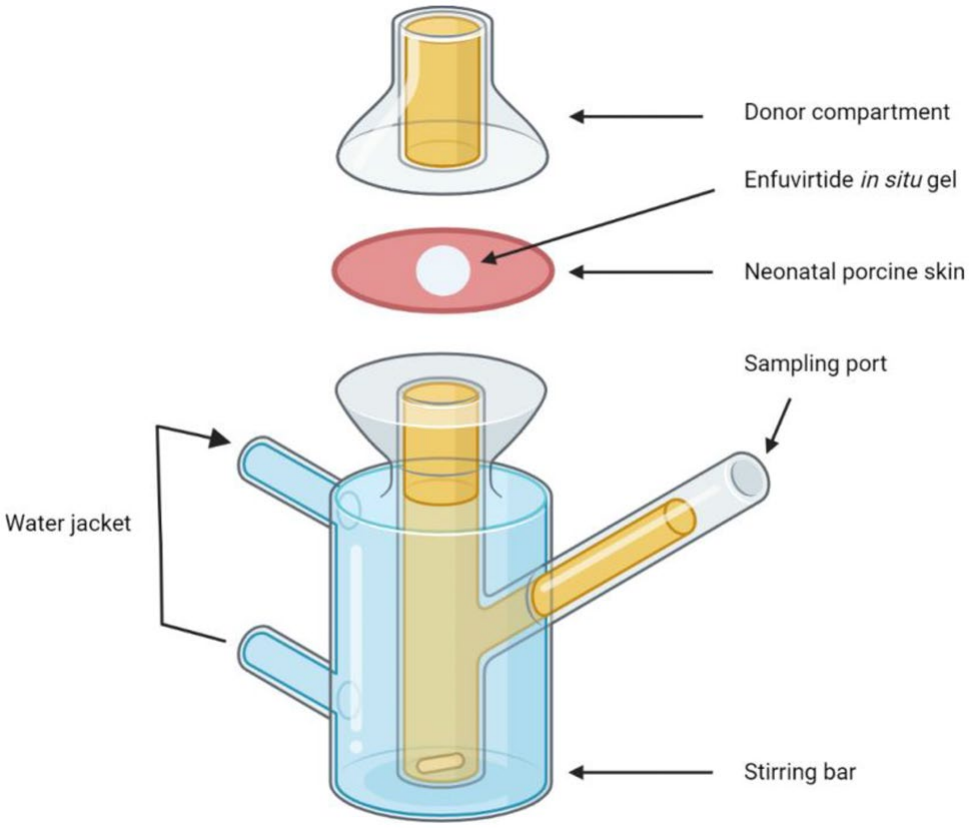
Schematic illustration of Franz-cell diffusion system for in vitro permeation studies of enfuvirtide loaded in thermosensitive in situ forming gel [31]

The Franz-cell diffusion model was used to assess the delivery of the in situ forming gel in vitro, with the apparatus depicted in Fig. 2. A solution was prepared by adding 170 mg of poloxamer 407 to 400 mg of deionised water and was kept in the fridge over night for swelling. The injectable formulation encapsulated with enfuvirtide was prepared by blending the poloxamer 407, 10 mg of poloxamer 188, 20 mg of enfuvirtide and 400 mg of deionised water uniformly at 25 °C. An U-100 insulin syringe (BD Medical-Pharmaceutical Systems, Franklin, USA) was used to inject 50 µL of the formulation into full thickness neonatal porcine skin. The skin was then attached to the donor compartment using cyanoacrylate adhesive (TEX YEAR INDUSTRIES INC., Taipei, Taiwan). The receptor compartment contained 12 mL of prewarmed PBS (pH 7.4) and a magnetic stir bar (4 mm × 10 mm) was introduced at a rotation speed of 600 rpm to homogenise the dissolution of the compounds. The donor was mounted onto the top of the receptor compartment and was fixed in position with a spring clamp. To minimise medium evaporation and prevent contamination from the environment, Parafilm^®^ was used to seal the juncture of the two compartments and cover the top of the donor. A water jacket surrounded the receptor, maintaining the temperature of the system at 37 °C. At this temperature, poloxamers were capable of forming hydrogels through hydrophilic-hydrophobic interactions between ethylene oxide and propylene oxide within the polymer [30].

The receptor compartment of the diffusion model represents the dermal microcirculation beneath the skin; therefore, if samples removed from the receptor contain drug, it may suggest that drug can be delivered to the bloodstream. Samples extracted from the skin represent drug that remained in the skin [31]. Samples from the receptor were analysed by the HPLC system after being diluted appropriately with mobile phase and centrifuged to remove insoluble particles. Skin samples were cut into pieces and were added to 2-mL centrifuge tubes with 500 µL of deionised water and two metal beads. Subsequently, they were crushed with a TissueLyser LT (QIAGEN, Hilden, Germany) at 50 rpm for 15 min. Then, 500 µL of ACN was added to precipitate proteins and the tubes were centrifuged at 16,000 × g for 15 min, with 200 µL of supernatant removed for analysis.

### Selectivity and specificity

In order to ensure that enfuvirtide can be identified in the presence of other substances, excipients from the formulation, release media and neonatal porcine skin were employed as possible impurities. To assess chromatographic separation of enfuvirtide from the aforementioned interferents, blank samples of the skin, PBS (pH 7.4, 10 mM), excipients and samples containing each possible interference were spiked with 10 µg/mL of enfuvirtide, extracted and analysed as described in “Sample preparation”.

### Linearity and range

Calibration plots were prepared by assaying standard solutions containing eight different concentrations over 3 days for enfuvirtide. A calibration curve was constructed by plotting peak area on the *Y*-axis and the nominal concentration on the *X*-axis. The limit of detection (LOD) was calculated using Eq. (1) and the limit of quantification (LOQ) was calculated using Eq. (2), where *σ* was the standard deviation (SD) of the data response used to construct the regression line and *S* was the slope of the line [32, 33].

1 $$\mathrm{LOD}=\frac{3.3\ \partial}{\mathrm{S}}$$

2 $$\mathrm{LOQ}=\frac{10\ \partial}{\mathrm{S}}$$

### Accuracy and precision

Intra-day accuracy and precision were evaluated by determining the response of nine replicates of QC solutions which were prepared and injected on the same day. Moreover, inter-day accuracy and precision were evaluated by determining the response of three replicates of QC solutions which were prepared and injected on 3 consecutive days using three different calibration curves. Accuracy was determined by comparing the calculated concentrations to their theoretical values (%RE) and the precision was calculated as the percent coefficient of variation (%RSD).

### Stability of samples

A stock solution of 1 mg/mL enfuvirtide was prepared according to “Stock, calibration standard and quality control solution preparation” and diluted with an appropriate volume of PBS containing 1% w/v of poloxamer 188, and 17% w/v of poloxamer 407 in order to simulate the condition for the formulation, or mobile phase to mimic the sample after dilution. The stability samples were placed in glass vials with a final concentration of 10 µg/mL of enfuvirtide. Solutions were kept at ambient temperature, in the fridge (2–8 °C), in an incubator at 37 °C and in a freezer at −20 °C. Additionally, a series of solutions were covered with aluminium foil to investigate the effect of light on drug degradation. Every 24 h, 200 µL was removed from each vial over 14 consecutive days, or until the calculated concentration was below 85% of the original concentration (*d*_0_) [12]. The stability of samples after 2 freeze-thaw cycles was evaluated. All samples were prepared in triplicate and analysed using the validated HPLC method to evaluate the recovery.

### Adsorption study

**Fig. 3:**
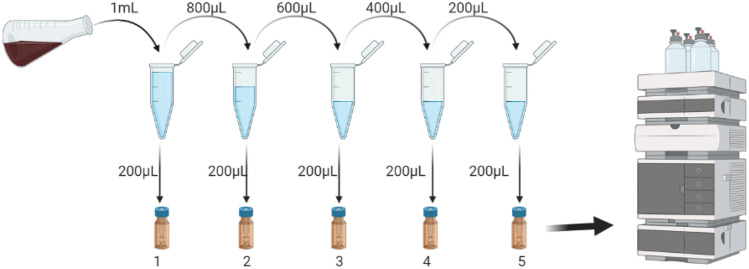
Transfer process of samples for adsorption study

Adsorption to the container and filter membrane is a common phenomenon due to the specific characteristics of peptide drugs, such as ionisation and solubilisation properties. In order to minimise the loss of sample during preprocessing, recovery of transfer and filtration were examined. To assess this, a sample was transferred from one centrifuge tube (2 mL) to another, resulting in a total of five consecutive transfer steps. The samples were quantified using HPLC to assess the adsorption by pipette tips and vials used in instrumental analysis [10], as depicted in Fig. 3.

Mean recovery of enfuvirtide after processing was determined by comparing the peak area of the extracts from skin spiked with enfuvirtide (10 µg/mL) with the peak area obtained by injection of the same amount of drug into the HPLC system directly.

### Statistical analysis

Statistical analysis was conducted using Statistical Product and Service Solutions (SPSS) for Windows version 22. Analysis of variance (ANOVA) was used to evaluate the differences between groups and the significance level *p* value was set to 0.05. GraphPad Prism^®^ version 8.0 (GraphPad Software, San Diego, CA, USA) was used for analysis of regression.

## Results and discussion

### Linearity and limit of quantification

**Fig. 4:**
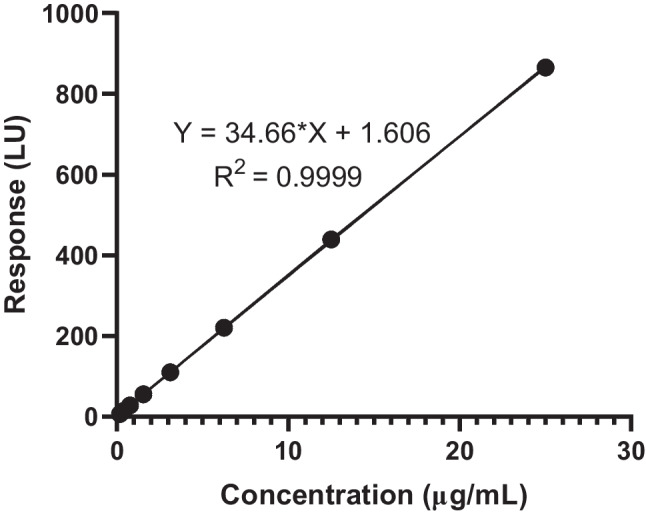
Calibration plot for HPLC fluorometric method to quantify enfuvirtide (means ± SD, *n* = 5)

There was a positive linear correlation between enfuvirtide concentration and assay signal over the entire validation range (0.19 to 25 µg/mL). The correlation coefficient (*R*^2^) for the calibration curve was 0.9999 and the calibration plot is shown in Fig. 4. The LOD and LOQ calculated according to ICH Q2(R1) were 0.24 µg/mL and 0.74 µg/mL, respectively. The LOQ was lower than the concentration of enfuvirtide detected from the in vitro transdermal study, confirming the method was sufficiently sensitive to detect drug in samples taken at early points.

### Accuracy and precision

The accuracy and precision were evaluated by observing the response of three QC samples in 1 day (intra-day) and within 3 consecutive days (inter-day). The precision was reflected by the percent of coefficient of variation (RSD%). The precision was obtained by the relative error (RE%) between the calculated value and theoretical value. Intra-day and inter-day accuracy for enfuvirtide in mobile phase (water:acetonitrile = 50:50, v/v) were within the range of ± 15%. Similarly, intra-day and inter-day precision were within the range of ± 15%, with further details listed in Table 2.

**Table 2:** Intra-day and inter-day accuracy and precision (means ± SD, *n* = 3)

	**Day**	**Theoretical concentration (μg/mL)**	**Experimental concentration (μg/mL)**	**Precision (RSD%)**	**Accuracy (RE%)**
		25.00	27.91 ± 0.05	0.19	11.66
	1	6.25	6.99 ± 0.18	2.63	11.79
		1.56	1.59 ± 0.03	1.76	1.86
		25.00	27.49 ± 0.09	0.34	9.96
Inter-day	2	6.25	6.93 ± 0.20	2.91	10.84
		1.56	1.56 ± 0.05	3.08	−0.06
		25.00	27.96 ± 0.36	1.29	11.83
	3	6.25	6.77 ± 0.38	5.64	8.35
		1.56	1.60 ± 0.02	1.04	2.45
		25.00	27.31 ± 0.86	3.16	9.25
Intra-day		6.25	6.81 ± 0.13	1.90	9.03
		1.56	1.69 ± 0.09	5.39	8.09

### Specificity and selectivity

**Fig. 5:**
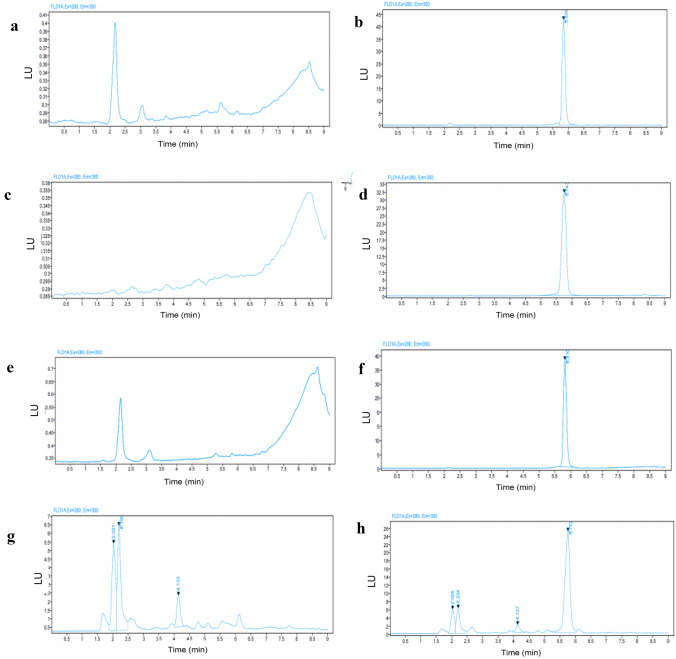
Representative chromatograms of enfuvirtide in release media combined with other components that could possibly exist in in vitro studies. **a** Blank release media. **b** Drug in release media. **c** Mobile phase. **d** Drug in mobile phase. **e** Mixture of gel formulation (poloxamer 188 and poloxamer 407). **f** Drug in the mixture of gel formulation (poloxamer 188 and poloxamer 407). **g** Mixture of porcine skin. **h** Drug in the mixture of porcine skin. LU stands for luminescence units

Specificity and selectivity of the method were assessed by analysing blank samples with potential interference from the mobile phase, release media and pig skin, and blank samples spiked with interference and enfuvirtide. As presented in Fig. 5, with the addition of mobile phase, release media, formulation excipients and the porcine skin, no interference peaks were found around the retention time of enfuvirtide, confirming that the assay has appropriate specificity and selectivity for enfuvirtide quantification [26].

### Stability of samples

Stability of enfuvirtide (10 µg/mL) in PBS and mobile phase was assessed within the conditions that have been studied, including 37 °C, 37 °C protected from light, room temperature, room temperature protected from light, 4 °C, 4 °C protected from light and −20 °C freeze-thaw cycles. A sample of 200 µL was analysed from each storage environment every 24 h until the recovery was beyond the range of 85~115%, with the stability investigation lasting for a maximum of 14 days. Restricted to the hydration property of the skin, in vitro transdermal studies should be carried out no more than 2 days in order to keep the consistent barrier function of the skin. Hence, the stability of samples stored at 37 °C was explored for 2 days. Data obtained from the last sampling point in each condition is shown in Table 3.

**Table 3:** The calculated concentrations of the last stable sample under different conditions (means ± SD, *n* = 3)

**Conditions**	**In PBS**			**In mobile phase mixture**		
	**Day**	**Mean ± SD (µg/mL)**	**Recovery (%)**	**Day**	**Mean ± SD (µg/mL)**	**Recovery (%)**
37 °C	0	9.22 ± 0.10	92.19%	2	9.64 ± 0.06	96.44%
37 °C protected from light	2	8.62 ± 0.07	86.17%	2	9.67 ± 0.04	96.70%
Room temperature	0	9.73 ± 0.13	97.33%	14	9.65 ± 0.11	96.54%
Room temperature protected from light	2	8.94 ± 0.25	89.40%	14	10.21 ± 0.05	102.07%
4 °C	0	9.83 ± 0.33	90.28%	14	10.12 ± 0.09	101.18%
4 °C protected from light	2	9.82 ± 0.19	90.28%	14	9.55 ± 0.04	95.51%
2 Freeze-thaw cycles (−20 °C)	30	8.76 ± 0.09	87.64%	30	9.98 ± 0.03	99.79%

Displaying higher recovery after 14 days under various conditions, samples diluted with mobile phase were confirmed to be more stable compared with samples diluted with PBS, indicating that the addition of acetonitrile maintained the stability of the peptide [10, 34]. Light and 37 °C temperature could accelerate the degradation of enfuvirtide in PBS extensively, with less than 85% recovery after 24 h for samples without protection from light. This result was in good agreement with the specification of enfuvirtide which suggested that after reconstitution enfuvirtide should be refrigerated and used within 24 h [14]. The degradation of enfuvirtide in vials protected by aluminium foil was significantly slower than the samples exposed to light within 24 h (*p* < 0.05). No significant difference was found between foil-covered and uncovered vials in the presence of acetonitrile. Overall, these findings suggest that there was a need to use aluminium foil to shelter the drug from light in the in vitro study. Moreover, based on the results of stability studies of enfuvirtide, samples could be diluted with acetonitrile and stored at 4 °C or −20 °C prior to HPLC analysis.

### Adsorption study

**Fig. 6:**
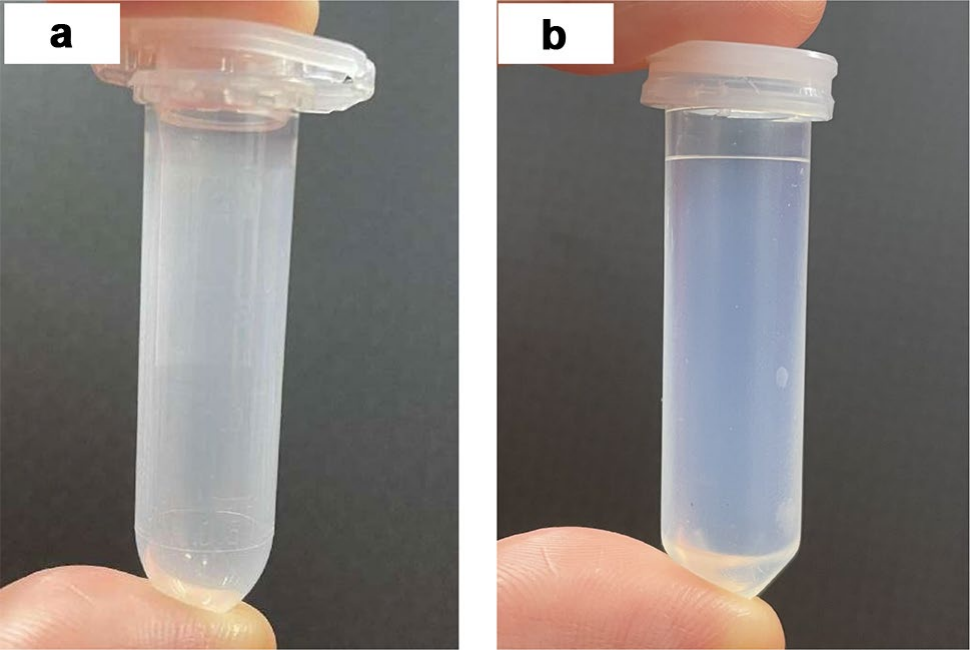
Centrifuge tubes made of PP (**a**) and PFA (**b**)

**Fig. 7:**
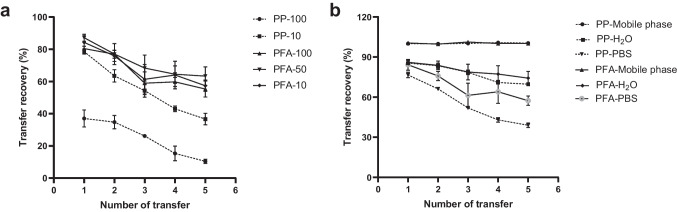
Effect of drug concentration (**a**) and solvent (**b**) on drug recovery after being centrifuged using tubes made of PP and PFA (means ± SD, *n* = 3). PP-10 and PP-100 indicated 10 µg/mL and 100 µg/mL of enfuvirtide in PP-made centrifuge tubes. PFA-10, PFA-50 and PFA-100 indicated 10 µg/mL, 50 µg/mL and 100 µg/mL of enfuvirtide in PFA-made centrifuge tubes. PP-mobile phase, PP-H_2_O and PP-PBS indicated 10 µg/mL of enfuvirtide prepared by mobile phase, H_2_O and PBS in PP-made centrifuge tubes. PFA-mobile phase, PFA-H_2_O and PFA-PBS indicated 10 µg/mL of enfuvirtide prepared by mobile phase, H_2_O and PBS in PFA-made centrifuge tubes

In order to remove insoluble particles or proteins and protect the HPLC system from blocking, centrifugation and filtration were indispensable during the extraction of drugs from the hydrogel and skin. For centrifugation, it was reported by Antonio et al*.* and Lawless et al. that chemical reactions between centrifuge tubes and enfuvirtide would occur when tubes made of poly (propylene) (PP) were used; hence, centrifuge tubes made of PTFE were employed as a replacement [35]. In this study, 2-mL centrifuge tubes made of PP and poly (fluoroalkoxy) (PFA) (Savillex, Eden Prairie, USA) were trialled as displayed in Fig. 6. As shown in Fig. 7a, the recovery decreased steadily following an increase in the number of transfers whether the tube was made of PP or PFA. Interestingly, compared with 10 µg/mL of enfuvirtide, less recovery was observed in 100 µg/mL of enfuvirtide with increasing number of transfers made from PP-centrifuge tubes (*p* < 0.05). The higher the concentration of the drug, the lower the recovery following transfer, indicating that PP-drug adsorption might be drug concentration related. The same tendency was not obvious in the case of PFA; however, overall, the recovery decreased with increasing numbers of transfer and was not significantly related to concentration (*p* > 0.05). To investigate the effect of solvent on adsorption, the same 10 µg/mL of enfuvirtide was prepared with mobile phase, H_2_O and PBS. According to Fig. 7b, in contrast with H_2_O and PBS, the addition of acetonitrile as an organic modifier presented 100% recovery both in PP and PFA tubes. Consequently, PP tubes were practicable for sample treatment in the study with the addition of acetonitrile.

**Fig. 8:**
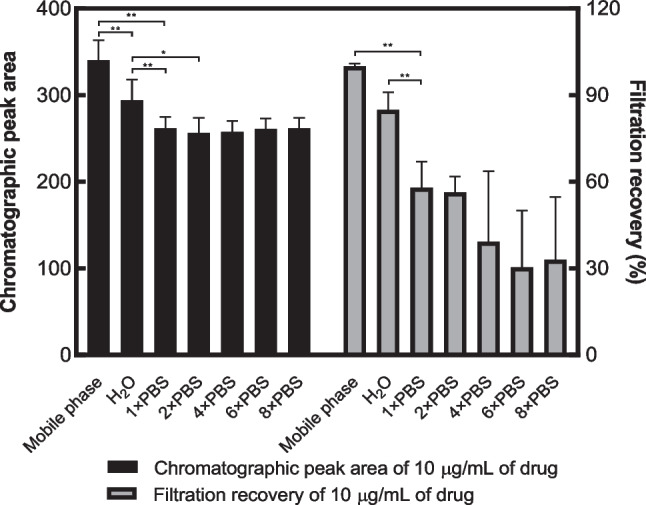
Effect of solvent on signal and filtration recovery (means + SD, *n* = 3). 1 × PBS (pH 7.4, 10 mM), 2 × PBS (pH 7.4, 20 mM), 4 × PBS (pH 7.4, 40 mM), 6 × PBS (pH 7.4, 60 mM), 8 × PBS (pH 7.4, 80 mM)

It is important to highlight that samples of the same concentration prepared with different solvents have different responses on the instrument, shown in Fig. 8. Samples diluted with mobile phase had the highest signal which was significantly different from samples prepared with deionised water and PBS of different ion concentrations (*p* < 0.001). As for filtration, a variety of filters made of different filter membranes were trialled in this study. There was an extensive loss of drug after filtration with nylon, cellulose acetate and poly (vinylidene fluoride) (PVDF), with a mean recovery of 5.66%, 25.16% and 14.78%, respectively (*n* = 3). For filters made of PTFE, approximately 100% drug remained after filtration when acetonitrile was used as the solvent, markedly higher than other filters (*p* < 0.05). As a result, PTFE filters were used in later studies.

### Application of developed method

**Fig. 9:**
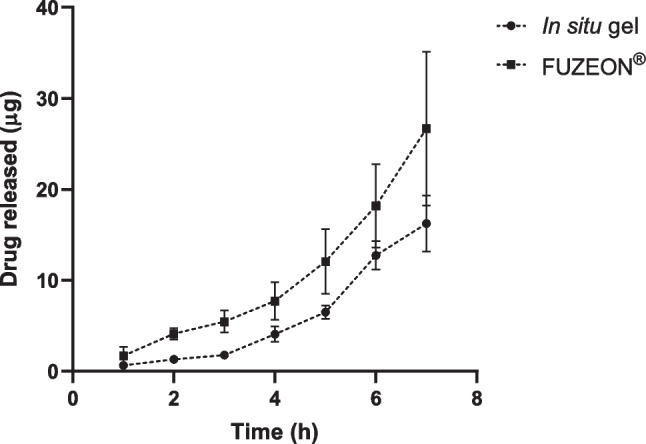
In vitro release of enfuvirtide across full thickness neonatal porcine skin from in situ forming gel and pure drug depot (FUZEON^®^) in 8 h (means ± SD, *n* = 3)

**Fig. 10:**
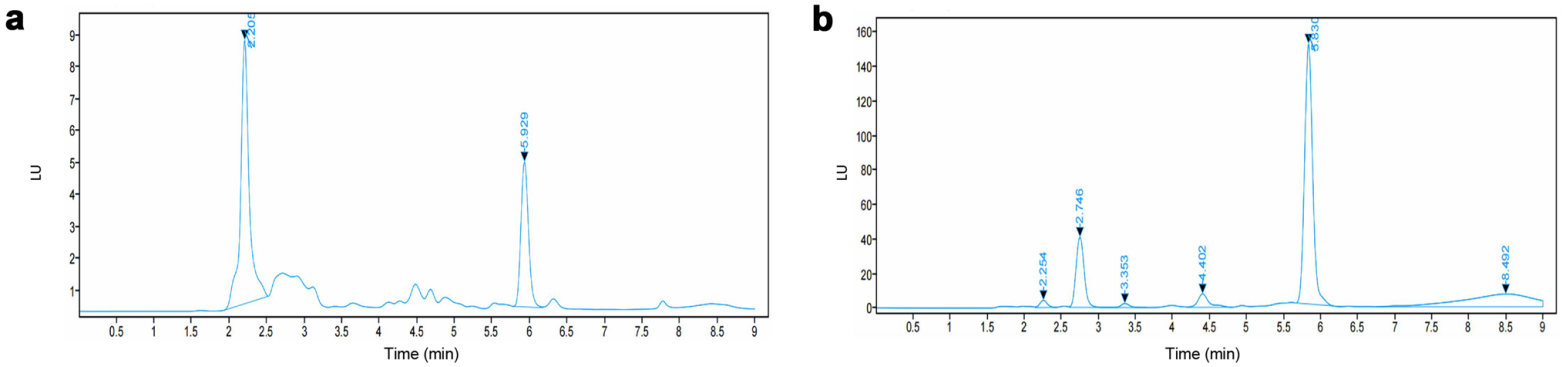
Representative chromatograms of sample analysis. **a** Drug release from the in situ forming gel after 1 h. **b** Drug remaining in skin following extraction after 7 h

The developed method was applied to quantify enfuvirtide delivered from the thermosensitive in situ gel following a Franz-cell diffusion experiment. Each 50 μL of formulation contained 72.69 μg drug. The amount of enfuvirtide delivered to the receptor compartment was assessed at predetermined times, 1, 2, 3, 4, 5, 6 and 7 h as shown in Fig. 9. Drug remaining in the skin was extracted and analysed using the method developed in this study, as shown in Fig. 10.

The control group, in which only the enfuvirtide solution was injected into the skin, did not display any sustained release of enfuvirtide, with 26.68 ± 10.45 μg being released and 45.31 ± 17.69 μg within the skin after 7 h. By comparison, the formation of the hydrogel entrapping the peptide within resulted in a sustained release profile, with 16.25 ± 7.08 μg in the receptor and 52.71 ± 17.55 μg in the skin after 7 h. In future studies, the formulation of the thermally responsive gel could be rationalised according to the result from in vitro studies in order to achieve a prolonged delivery.

### Comparison between the current and previous methods for quantification of enfuvirtide

The published methods for the detection of enfuvirtide in plasma are summarised in Table 4. Internal standards, such as deuterium-labelled enfuvirtide (d_60_-enfuvirtide), detergent (n-nonyl-β-d-glucopyranoside) and insulin, were used to avoid or minimise the loss of analytes, which increased the cost of these assays. In this study, introducing acetonitrile as an organic modifier to prepare a standard solution resulted in desirable response at low concentrations and yielded appropriate linearity (*R*^2^ = 0.9999). In addition, the recovery experiment confirmed that ordinary centrifuge tubes fabricated from PP were practicable for the use of sample processing, rather than using centrifuge tubes composed of PTFE, as reported by D’Avolio et al. [23] and Lawless et al. [29].

**Table 4:** A summary of methods for enfuvirtide quantification

**References**	**Instrument**	**Mobile phase**	**Run time (min)**	**Injection volume (µL)**	**Linear range**	***R***^**2**^	**Matrix**	**Internal standard**
This study	HPLC-FLD	Acetonitrile, 0.1% (v/v) phosphoric acid in water	9	30	0.74–25 µg/mL	0.9999	Skin	-
D’Avolio et al. [23]	HPLC-FLD	Water + 0.1% trifluoroacetic acid + 0.5% arginine hydrochloride, acetonitrile: water [70:30] + 0.1% trifluoroacetic acid + 0.5% arginine hydrochloride	16	50	0.078–10 µg/mL	0.999	Plasma	Insulin
Lawless et al. [29]	HPLC-FLD	0.1% TFA + 1% arginine hydrochloride, 70% ACN + 30% water	30	-	0.1–4 µg/mL	0.9961	Plasma	n-Nonyl-β-d-glucopyranoside
van den Broek et al. [24]	LC–MS/MS	0.25% (v/v) formic acid in water, 0.25% (v/v) formic acid in acetonitrile	11	-	0.02–10 µg/mL	0.9868 to 0.9993	Plasma	d_60_-enfuvirtide
van den Broek et al. [26]	LC–MS/MS	0.25% (v/v) formic acid in water, 0.25% (v/v) formic acid in methanol	13	-	0.1–10 µg/m	0.9929 ± 0.0081	Plasma	d_60_-enfuvirtide

In this study, separation of enfuvirtide was performed on a reversed-phase column with the elution of acetonitrile and 0.1% (v/v) phosphoric acid in deionised water, resulting in optimised chromatographic conditions to obtain desirable resolution in a short time. Compared with the mobile phase that previous HPLC fluorometric methods used, the mobile phase composition of this method was uncomplicated with no specific arginine hydrochloride contained. Moreover, a 30 μL sample volume and a run time of 9 min provided advantages for enfuvirtide analysis over other methods. The applicability of this method for the quantification of enfuvirtide from an in situ gel has been investigated.

## Conclusion

In this study, a HPLC fluorometric method has been developed and validated for the determination of enfuvirtide. Through the addition of acetonitrile, the adsorption of enfuvirtide to the container and filter was minimised, exhibiting desired linearity and recovery after sample processing. This method was confirmed to be efficient, inexpensive, highly accurate and specific. With a run time of 9 min and devoid of any internal standard, the validated method has demonstrated its effectiveness in the analysis of samples from a thermosensitive in situ forming gel in an in vitro study. After optimisation of the chromatography conditions, the drug peak was successfully separated from skin matrix. Therefore, this method is ready to be used as a reliable tool for the formulation rationalisation of enfuvirtide for intradermal delivery.

## Data Availability

Yes.
